# Expression of Membranous CD155 Is Associated with Aggressive Phenotypes and a Poor Prognosis in Patients with Bladder Cancer

**DOI:** 10.3390/cancers14061576

**Published:** 2022-03-19

**Authors:** Kohei Mori, Kazumasa Matsumoto, Noriyuki Amano, Dai Koguchi, Soichiro Shimura, Masahiro Hagiwara, Yuriko Shimizu, Masaomi Ikeda, Yuichi Sato, Masatsugu Iwamura

**Affiliations:** Department of Urology, Kitasato University School of Medicine, 1-15-1 Kitasato Minami-ku Sagamihara, Sagamihara 252-0374, Kanagawa, Japan; mori2020@med.kitasato-u.ac.jp (K.M.); dm19002@st.kitasato-u.ac.jp (N.A.); dai.k@med.kitasato-u.ac.jp (D.K.); sou-telb89@live.jp (S.S.); m_hagi_9103@yahoo.co.jp (M.H.); yulico@med.kitasato-u.ac.jp (Y.S.); ikeda.masaomi@grape.plala.or.jp (M.I.); sato.yuichi@kobal.co.jp (Y.S.); miwamura@med.kitasato-u.ac.jp (M.I.)

**Keywords:** poliovirus receptor, CD155, immunohistochemistry, bladder cancer, cystectomy, urothelial carcinoma

## Abstract

**Simple Summary:**

The presence of the poliovirus receptor (known as cluster of differentiation 155 (CD155)) in human cancer is an unfavorable prognostic marker. We investigated whether CD155 expression, divided into membranous and cytoplasmic types, affects the prognosis of bladder cancer (BC) in terms of recurrence-free survival (RFS) and cancer-specific survival (CSS). We found that membranous CD155 (mCD155)-positive cases had shorter periods of both RFS and CSS, whereas there was no association between cytoplasmic CD155 (cCD155) and survival outcomes. The results showed that CD155, especially mCD155, may serve as a poor prognostic marker in BC.

**Abstract:**

Objective: To investigate the relationship between clinicopathological findings and membranous CD155 (mCD155) or cytoplasmic CD155 (cCD155) expression in bladder cancer (BC). Methods: We retrospectively analyzed 103 patients with BC who underwent radical cystectomy between 1990 to 2015 at Kitasato University Hospital. Immunohistochemical staining was performed to evaluate CD155 expression in tumor cells. Cases with > 10% expression on the membrane or cytoplasm of tumor cells were positive. The Fisher′s exact test was used for categorical variables and the Kaplan–Meier method was used for survival outcomes. Univariate and multivariate Cox regression hazard models were used to evaluate the survival risk factors. Results: Cases that were mCD155-positive were associated with high-grade tumors (*p* = 0.02), nodal status (*p* < 0.01), and pT stage (*p* = 0.04). No association with any clinicopathological factor was observed in the cCD155 cases. Kaplan–Meier analysis showed that mCD155-positive cases had shorter periods of recurrence-free survival (*p* = 0.015) and cancer-specific survival (*p* = 0.005). Only nodal status was an independent predictor for both cancer-specific survival and recurrence-free survival in multivariate analysis (*p* = 0.02 and *p* < 0.01, respectively). Conclusion: mCD155 expression may be a marker of an aggressive phenotype and a poor prognosis in patients with BC.

## 1. Introduction

Bladder cancer (BC) is the tenth most common cancer worldwide, with approximately 550,000 new patients annually [1]. Japanese men have the highest incidence rates and Japanese women have the second highest incidence rates in Central and Eastern Asia [2] (age-standardized rate = 9.6 and 2.2 per 100,000, respectively). Radical cystectomy (RC) and pelvic lymph node dissection are indicated for patients with Bacillus Calmette-Guerin refractory to non-muscle invasive BC and those with muscle-invasive BC [3]. Unfortunately, approximately 40% of patients experience metastases to other organs or lymph nodes within five years after RC [4].

To improve the prognosis of patients who underwent RC, the introduction of maintenance chemotherapy [5] or immune checkpoint inhibitors [6] has been considered; however, not all patients with BC respond well to these anticancer drugs and treatment-associated adverse events have been observed. In addition, there is no biomarker for selecting patients who need additional treatments after RC in the early phase of disease.

Cluster of differentiation 155 (CD155, also known as nectin-like molecule family 5) is a transmembrane protein that was initially identified as a poliovirus receptor (PVR). CD155 is rarely expressed in most normal tissues, but is overexpressed in various carcinomas such as breast cancer [7,8], lung cancer [9,10], ovarian cancer [11], and prostate cancer [12]. Recently, Zhang et al. [13] showed by immunohistochemistry that CD155 is a robust prognostic factor of muscle invasive BC. Human cells express both membranous CD155 (mCD155) and cytoplasmic CD155 (cCD155) encoded by splicing isoforms of CD155; therefore, it is necessary to examine the expression of CD155 according to their localization.

Programmed death-ligand 1 (PD-L1) is well-known as an immune checkpoint protein. Some studies have reported anti-tumor effects with the use of both PD-L1 and CD155. Ma et al. [14] generated a bispecific antibody that targets both CD155 and PD-L1, and reported that T cell activities were synergically enhanced by both CD155 and PD-L1 blockage. Mao et al. [15] reported that blocking both CD155 and PD-L1 inhibited tumor cell proliferation and promoted the generation of effector T cells by using the transgenic mouse model.

In this study, we separately investigated the expression of mCD155 and cCD155 in BC cells. We also investigated the relationship between the expression of CD155 and PD-L1.

## 2. Materials and Methods

### 2.1. Patients and Data Collection

The clinical data of 143 consecutive patients with BC who underwent RC with pelvic and iliac lymphadenectomy from 1990 to 2015 at Kitasato University Hospital (Kanagawa, Japan) were retrospectively reviewed. We excluded 40 patients for the following reasons: 10 patients who had histological variants, including 3 with squamous cell carcinoma, 3 with adenocarcinoma, and 4 with small-cell carcinoma; 15 patients who had been previously treated with neoadjuvant chemotherapy (NAC); and 15 patients who were lost to follow-up. The patients′ characteristics were obtained from their medical records including age at surgery, sex, pathological status including pT stage and pN stage, grade, presence of lymphovascular invasion (LVI), history of AC, and history of salvage chemotherapy (SC). Tumor grade was assessed according to the 1973 World Health Organization grading system and the 2002 TNM Classification of Malignant Tumors. AC was performed for patients with >pT3 or those with positive lymph node status. All patients who received AC or SC also received platinum-based chemotherapy. The chemotherapeutic response was evaluated by the Response Evaluation Criteria in Solid Tumors (RECIST) version 1.1. We categorized the patients as either responder (complete or partial response) or non-responder (stable disease or disease progression).

### 2.2. Immunohistochemistry for CD155

Formalin-fixed paraffin-embedded 3-μm thick sections of harvested samples were deparaffinized in xylene, rehydrated in a series of decreasing ethanol concentration, and treated with 3% hydrogen peroxide for 10 min. Tissues were subjected to antigen retrieval by incubation with 0.01 mol/L citrate buffer (pH 6.0) in 0.1% Tween 20 at 121 °C for 10 min. After cooling to room temperature (RT) and blocking in 0.5% casein for 10 min, the sections were incubated with anti-CD155 monoclonal antibody (1:200; Cusabio Technology LLC, Houston, TX, USA) for 2 h at RT. After rinsing in Tris-buffered saline (0.01 M Tris-HCl pH 7.5, 150 mM NaCl) three times for 5 min each, the sections were incubated with horseradish peroxidase (HRP)-labeled polymer reagent (EnVision + Dual Link System HRP Kit; Dako, Glostrup, Denmark) for 30 min at RT. The sections were finally visualized with a stable DAB solution (Invitrogen, Carlsbad, CA, USA) and counterstained with Mayer′s hematoxylin. The expression of mCD155 and cCD155 in tumor cells was separately considered. The staining of vascular endothelial cells was used as an internal positive control. A result showing more than 10% of tumor cells with the same level or stronger membranous or cytoplasmic staining compared with vascular endothelial cells was considered positive. All of the immunostained sections were reviewed by two investigators (KM and YS) without any knowledge of the clinical data. Discordant cases were reviewed and discussed until a consensus was reached. We also compared the staining of CD155 and PD-L1 as previously described [16], using the same cohort. PD-L1 expression was evaluated separately for tumor cells and tumor-infiltrating lymphocytes (TILs). PD-L1 positivity was defined as more than 5% of positive tumor cells or moderate to markedly positive TILs.

### 2.3. Statistical Analyses

Age (<65 vs. ≥65 years), tumor stage (≤pT2 vs. ≥pT3), grade (grade 1 and 2 vs. grade 3), and nodal status (N0 vs. N1 and 2) were evaluated as dichotomized variables. The expression of PD-L1 was examined in parts of all specimens (n = 67). The association between CD155 expression and clinicopathological characteristics was examined by the Fisher′s exact test. Cancer-specific survival (CSS) and recurrence-free survival (RFS) were estimated by the Kaplan–Meier method and the log-rank test. Univariate and multivariate analyses were performed to estimate the association between CD155 expression and pathological stage and grade, presence of LVI, and nodal status with the Cox proportional hazards regression model. All statistical computations were conducted with JMP^®^15 for Windows (SAS Institute Inc., Cary, NC, USA). Statistical significance was determined to be *p* < 0.05.

## 3. Results

### 3.1. Immunohistochemistry Analysis of CD155

**Figure 1:**
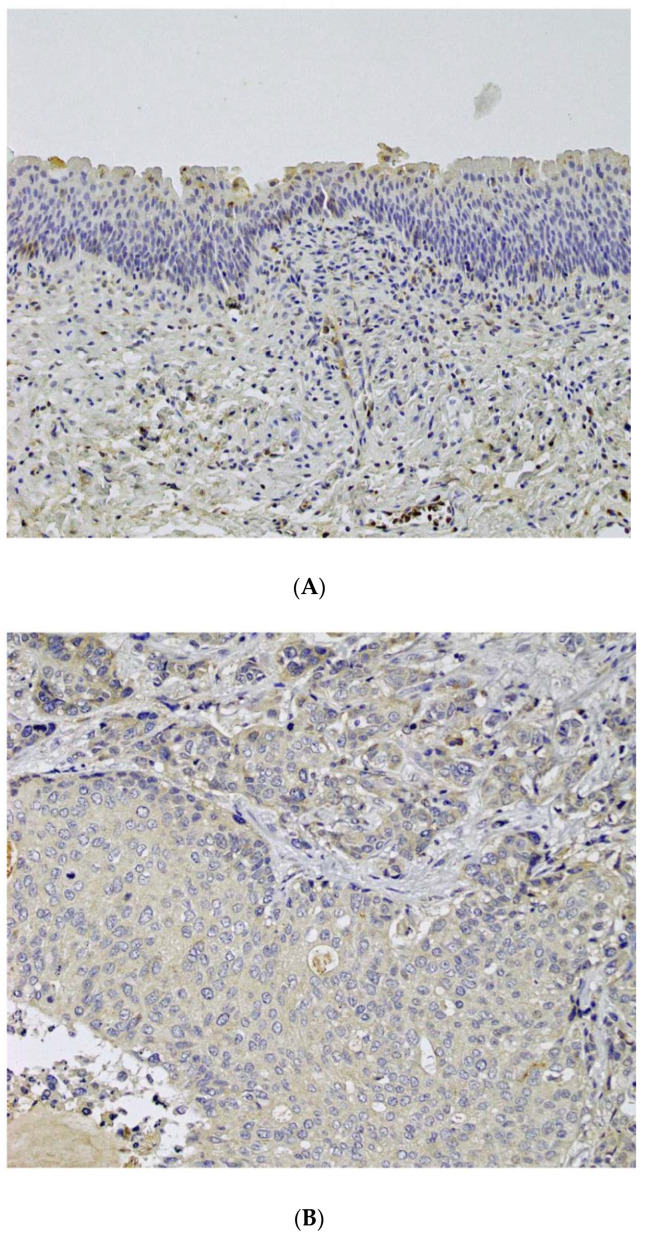

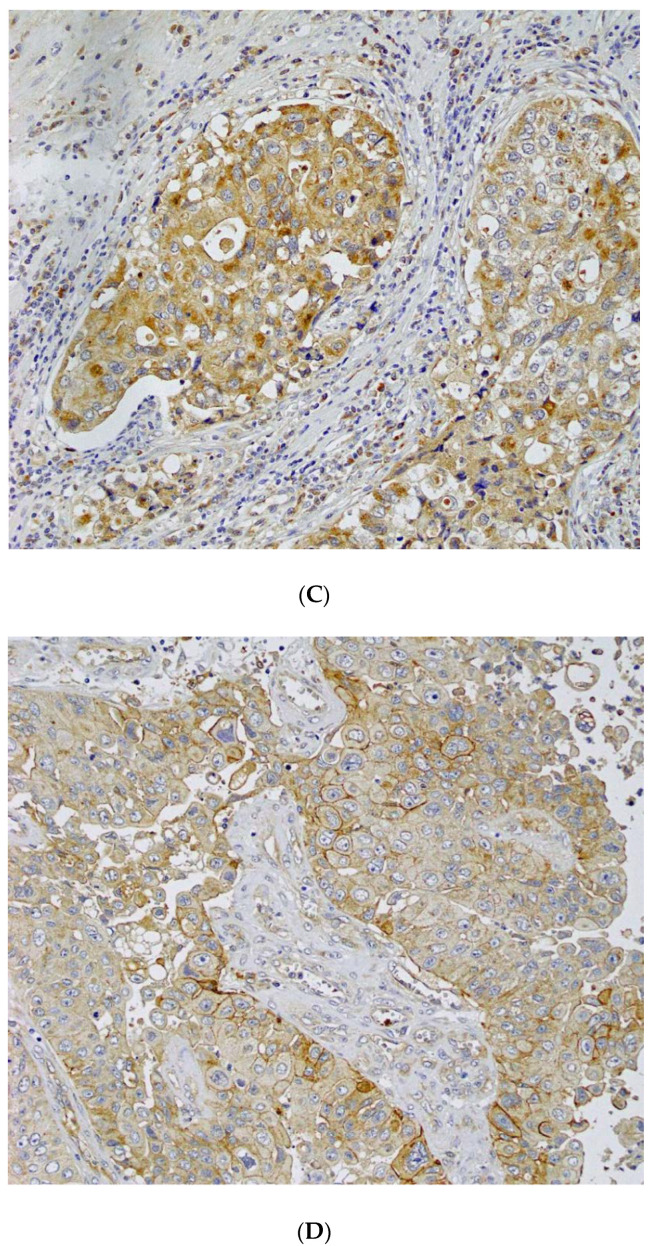
CD155 expression in non-neoplastic urothelial cells and urothelial carcinoma. (**A**) No or weak staining was observed in the non-neoplastic urothelial cell; (**B**) negative staining; (**C**) cytoplasmic staining; (**D**) membranous staining in urothelial carcinoma cells (original magnification, 400×).

Representative photomicrographs of normal urothelial and carcinoma tissues stained for CD155 are illustrated in Figure 1. Normal urothelial tissue showed negative to weak immunoreactivity to CD155 (Figure 1A). CD155 staining was observed in the cytoplasm and membrane of tumor cells (Figure 1C,D).

### 3.2. Association of Clinicopathological Characteristics with CD155 Expression

Table 1 summarizes the clinicopathological characteristics of all patients and their association with mCD155 and cCD155 expression. Age at cystectomy, sex, presence of carcinoma in situ, and lymphovascular invasion did not differ between the mCD155 and cCD155 expression groups. mCD155 positivity was associated with pT stage, pathologic grade, and nodal status (*p* = 0.04, 0.02, and < 0.01, respectively). Thirteen patients received SC, and no significant difference was observed between the responders and non-responders in both CD155 expression groups (*p* = 0.71 and 0.56, respectively). A significant difference was also not observed in recurrence status in both CD155 expression groups (*p* = 0.09 and 0.49, respectively). The expression of mCD155 and cCD155 was not associated with PD-L1 expression in both tumor cells and TILs.

### 3.3. Association of Survival Outcomes with CD155 Expression

**Figure 2:**
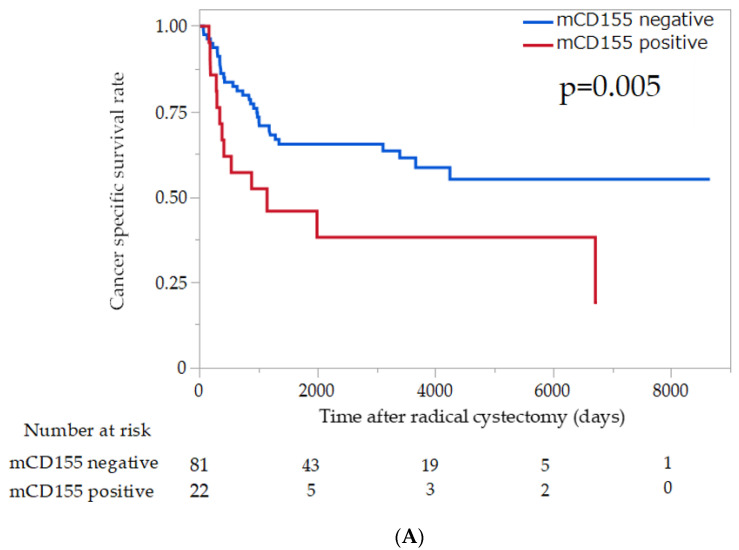

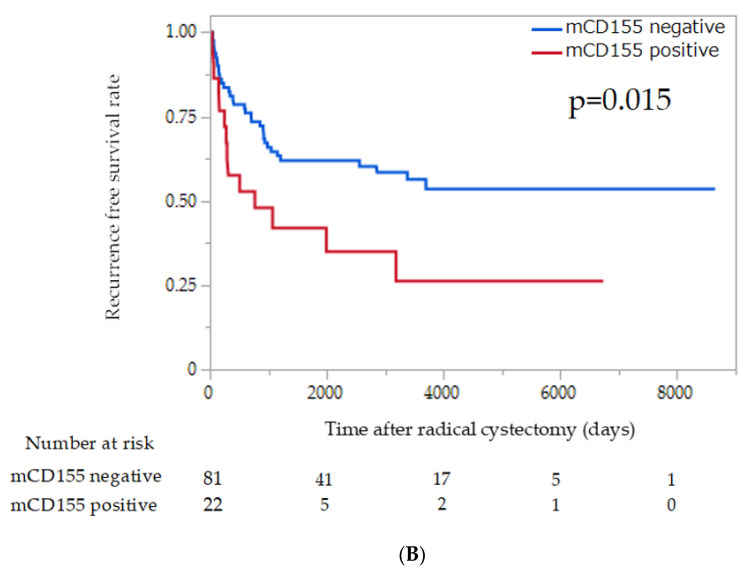
Probability of survival in patients with urothelial carcinoma of the bladder according to mCD155 expression estimated using the Kaplan–Meier method. (**A**) Cancer-specific survival; (**B**) recurrence-free survival.

Kaplan–Meier analysis showed that mCD155 positivity was associated with a significantly increased risk of disease recurrence and cancer-specific death (*p* = 0.015 and 0.005, respectively; Figure 2). There was no significant difference between cCD155 expression and prognosis (*p* = 0.34 and 0.32, respectively; Supplementary Figure S1). Univariate Cox regression analysis showed that mCD155 positivity, nodal status, pathological stage, and T stage were significantly associated with CSS and RFS, respectively (Table 2). Multivariate Cox regression analysis revealed that only nodal status was an independent prognostic factor for RFS and CSS (Table 2).

## 4. Discussion

CD155 was initially identified as a poliovirus receptor [17] in humans; recent discoveries have revealed greater insights into both its structure and its function. CD155 is classified as membrane CD155, which is expressed on the cytoplasmic membrane of tumor cells; and cytoplasmic CD155, which is expressed in the cytoplasm of tumor cells. Both types are expressed in human cells. CD155 is rarely expressed in most normal tissues, but is overexpressed in various carcinomas [7,8,9,10,11,12]. In terms of BC, Cong et al. [18] analyzed 797 patients from The Cancer Genome Atlas and Genome Expression Omnibus to establish a relationship between the mRNA expression of CD155 and the prognosis of several cancer types including BC. Zhang et al. [19] investigated 228 BC patients who underwent RC and reported that CD155 protein was significantly overexpressed on the membrane of tumor cells compared to matched normal urothelial cells. The current study was the first to use immunohistochemistry to investigate CD155 expression and localization in patients with BC who underwent RC separately for mCD155 and cCD155.

This study showed that the expression of mCD155 was significantly correlated with pathological stage, grade, and lymph node metastasis, suggesting that mCD155 overexpression in BC may act as a biologically aggressive factor by playing critical roles in tumor cell migration and proliferation. Minami et al. [20] reported that CD155 has a critical role in integrin Alpha-v beta-3 clustering and focal complex formation, potentially leading to cell migration. CD155 also affects tumor cell proliferation. Kakunaga et al. [21] showed that CD155 enhances growth factor-induced activation of Ras/Raf/mitogen-activated protein kinase/extracellular signal-regulated kinase signaling, leading to the upregulation of cell cycle regulators such as cyclins D2 and E, and eventually shortening the G1 phase of the cell cycle, thereby promoting tumor cell proliferation. By contrast, no clinicopathological factors were correlated with cCD155 expression in the present study. Okumura et al. [13] showed that mCD155 promoted the CD226-mediated cytotoxic activity of natural killer cells, whereas cCD155 inhibited this activity in a mouse model of melanoma. Although their findings showed that the expression of cCD155 was a poor prognostic factor, there have been no reports on the association between cCD155 expression and clinicopathological findings, nor have there been reports on the correlation between soluble CD155 levels in the blood and cCD155 expression levels in tumor cells. The roles of mCD155 and cCD155 in the tumor environment might differ depending on the type of malignancy and the environment; however, this correlation is not clearly understood.

Regarding the correlation between PD-L1 and CD155, Wang et al. [22] reported that breast cancer patients with positive CD155 expression have a higher percentage of CD4+/PD-L1 + TILs than those with negative CD155 expression. One possible reason is that the induction of interferon gamma secretion by activation of toll-like receptor 4 occurs through the same pathway as the upregulation of CD155 expression during the activation process of T cells in the tumor immune microenvironment. Chen et al. [23] investigated the expression of CD155 and PD-L1 in triple-negative breast cancer cell lines, and showed that activation of the CD155 pathway caused resistance to anti-PD-L1 treatment. The authors also demonstrated that triple-negative breast cancer cells co-expressed higher levels of CD155 and PD-L1 than non-triple-negative breast cancer cells. However, Smazynski et al. [11] reported that CD155 expression was not significantly related to the expression of PD-L1 in high-grade serous ovarian cancer. They also examined the correlation between the expression between CD155 and PD-L1 in several types of cancer cells, including BC, and concluded that no cancer showed a significant relationship. In the present study, neither mCD155 nor cCD155 was significantly related to PD-L1 in both tumor cells and TILs of BC, which supported previous reports. However, analysis on the molecular and gene expression levels is necessary to clarify the relationships. To understand the complex network of PD-L1 and CD155 signaling in tumor immunity, further studies will be needed to explore the dynamics of the association of CD155 with PD-L1, which will possibly contribute to the development of novel immunotherapies for the treatment of several cancer types.

The influence of CD155 on the tumor immune microenvironment is gradually becoming clear. Yu et al. [24] showed that T-cell immunoglobulin and immunoreceptor tyrosine-based inhibitory motif domain (TIGIT) can bind to CD155 with high affinity and suppress T cell activation indirectly by modulating dendritic cell activity. These findings indicate that high CD155 expression on tumor cells leads to tumor immune tolerance; as a result, the cancer cells easily increase and expand to other organs. A phase II study of the anti-TIGIT antibody tiragolumab was conducted in patients with PD-L1-positive non-small cell lung cancer [25]. Patients treated with tiragolumab plus atezolizumab showed significant improvements in objective response rate and progression-free survival compared to those treated with placebo plus atezolizumab. Attalla et al. [26] identified TIGIT as a possible target for monotherapy or combination therapy with other immune checkpoint inhibitors in patients with urothelial carcinoma. In the present study, there was no significant different between the response rate of SC and the expression of both mCD155 and cCD155. While chemotherapy was not enough to improve the survival in patients with CD155-positive BC, the efficacy of immune checkpoint inhibitors may be examined in these patients irrespective of PD-L1 expression. In addition, clinical trials of anti-TIGIT antibody are warranted for patients with CD155-positive BC. Currently, several clinical trials of atezolizumab plus tiragolumab in patients with cisplatin-ineligible muscle-invasive BC and those with locally advanced or metastatic urothelial carcinoma are ongoing [27].

This study had some limitations. First, it was a retrospective study. Second, the sample size was small, with only 22 and 24 patients in the mCD155 and cCD155 expression groups, respectively. Future large cohort studies are needed to confirm the results. Third, RC was performed by multiple surgeons and the selection of additional treatment such as AC and SC was decided by each doctor; these differences might have influenced the prognostic status. Finally, the relationship between CD155 and PD-L1 expression was restricted because only a limited section of the specimen was stained. However, the analysis of CD155 expression in RC specimens may explain the differences in prognosis.

## 5. Conclusions

The high expression of mCD155 in patients who underwent RC was associated with biologically aggressive factors and a poor prognosis. When anti-TIGIT antibody is available, CD155 may be a useful marker for predicting the prognosis and effects of novel treatments.

## Figures and Tables

**Table 1:** Association of mCD155 expression with clinical and pathological characteristics.

		CD155 Expression					
Characteristics		mCD155			cCD155		
	Total No.	negative	positive	*p*	negative	positive	*p*
Total (%)	103	81	22		79	24	
Age, years							
≤65	48	38	10	0.9	36	12	0.81
≥65	55	43	12		43	12	
SEX							
Male	86	69	17	0.35	66	20	0.98
Female	17	12	5		13	4	
Tumor stage							
≤pT2	52	45	7	0.04	39	13	0.82
≥pT3	51	36	15		40	11	
Pathologic grade							
Grade 1 and 2	32	30	2	0.02	25	7	0.81
Grade 3	71	51	20		54	17	
Carcinoma in situ							
Present	19	14	5	0.54	14	5	0.77
Absent	84	67	17		65	19	
LVI							
Present	54	39	15	0.22	38	16	0.08
Absent	37	31	6		32	5	
Nodal status							
pN0	76	65	11	<0.01	57	19	0.61
pN+	27	16	11		22	5	
PD-L1 expression in tumor cell							
Negative	54	43	11	0.47	43	11	0.83
Positive	13	9	4		10	3	
PD-L1 expression in TILs							
Negative	42	34	8	0.55	32	10	0.54
Positive	25	18	7		21	4	
Adjuvant chemotherapy							
Yes	14	11	3	0.98	10	4	0.73
No	89	71	18		69	20	
Salvage chemotherapy							
Responder	4	3	1	0.71	3	1	0.56
Non-responder	9	7	2		4	5	
Recurrence							
Yes	48	34	14	0.09	35	13	0.49
No	55	47	8		44	11	
Cancer-specific death							
No	63	50	13	0.81	52	11	0.09
Yes	40	31	9		27	13	

CD155, cluster of differentiation 155; mCD155, membranous CD155; cCD155, cytoplasmic CD155; LVI, lymphovascular invasion; PD-L1, programmed death-ligand 1; TIL, tumor-infiltrating lymphocytes.

**Table 2:** Univariate and multivariate Cox proportional hazards analyses to predict cancer-specific survival and recurrence free survival in patients with bladder cancer treated with radical cystectomy.

CSS (Cancer-Specific Survival)						
	Univariate			Multivariate		
	HR	95% CI	*p*-value	HR	95% CI	*p*-value
mCD155 positive	2.43	1.26–4.69	<0.01	1.35	0.65–2.79	0.42
pN+	2.94	0.16–5.41	<0.01	2.43	1.17–5.1	0.02
Grade3	2.08	1.01–4.27	0.04	1.1	0.51–2.42	0.81
LVI present	1.71	0.84–3.46	0.13	1.1	0.53–2.35	0.76
pT3-4	2.8	1.50–5.22	<0.01	1.79	0.85–3.74	0.12
**RFS (Recurrence-Free Survival)**						
	Univariate			Multivariate		
	HR	95% CI	*p*-value	HR	95% CI	*p*-value
mCD155 positive	2.13	1.14–4.01	<0.01	1.26	0.63–2.52	0.52
pN+	3.89	2.18–6.93	<0.01	3.6	1.73–7.44	<0.01
Grade3	1.92	0.98–3.77	0.04	1.43	0.68–3.14	0.34
LVI present	1.48	0.79–2.78	0.21	0.65	0.31–1.39	0.27
pT3-4	2.43	1.35–4.37	<0.01	1.78	0.99–3.95	0.05

CI, confidence interval; HR, hazard ratio; LVI, lymphovascular invasion; mCD155, membranous cluster of differentiation 155.

## Data Availability

The datasets used and/or analyzed during the study are available from the corresponding author on reasonable request.

## Supplementary Materials

The following are available online at https://www.mdpi.com/article/10.3390/cancers14061576/s1, Figure S1: Survival analysis using a Kaplan–Meier curve to determine recurrence-free survival and cancer-specific survival for positive and negative cCD155 expression.
