# Supplementary material for: Expression of Membranous CD155 Is Associated with Aggressive Phenotypes and a Poor Prognosis in Patients with Bladder Cancer
Source: Cancers (Basel). 2022 Mar 19;14(6):1576. doi: 10.3390/cancers14061576 (PMC8946612; doi:10.3390/cancers14061576)
Supplement: Supplementary file 1 [file cancers-14-01576-s001.zip › cancers-1612502-supplementary.pdf]

(**A**)

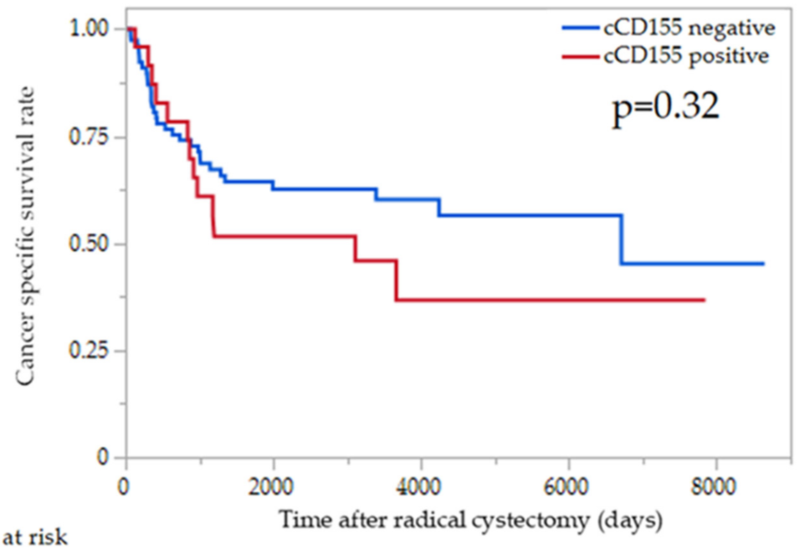

Number at risk

cCD155 negative	79	38	18	5	1
cCD155 positive	24	10	4	2	0

(**B**)

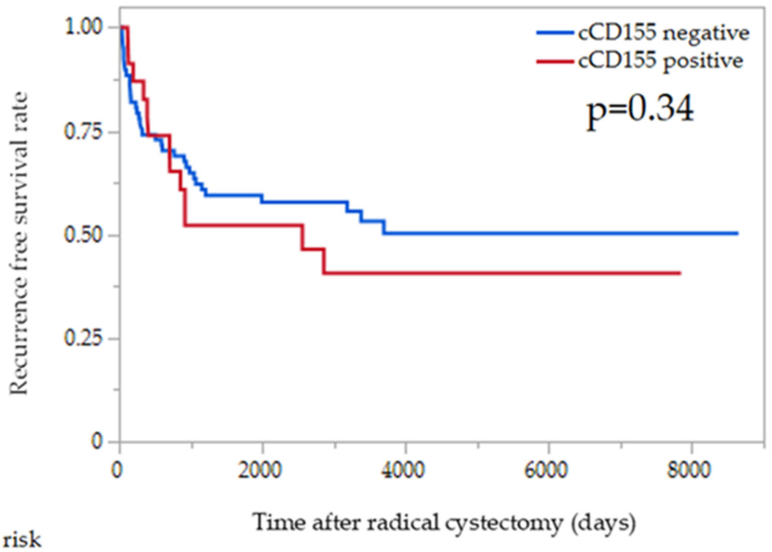

Number at risk

cCD155 negative	79	36	17	15	1
cCD155 positive	24	10	2	4	0

Figure S1. Probability of survival in patients with urothelial carcinoma of the bladder according to cCD155 expression estimated using the Kaplan–Meier method. (**A**) Cancer-specific survival, (**B**) Recurrence-free survival.
